# Effects of “On-Water” on the Free Radical Polymerization of Methacrylates

**DOI:** 10.1021/acs.langmuir.6c01890

**Published:** 2026-06-09

**Authors:** Yusaku Kambe, Shin-ichi Matsuoka

**Affiliations:** Division of Soft Materials, Department of Engineering, Graduate School of Engineering, Nagoya Institute of Technology,Gokiso-cho, Showa-ku, Nagoya, Aichi 466-8555, Japan

## Abstract

Water–organic interfaces, referred to as “on-water”, have recently attracted considerable attention because of their ability to accelerate various organic reactions. In this study, the effects of “on-water” interface on the conventional free radical polymerization of methacrylates were investigated. Polymerizations were carried out under various conditions, including variations in monomer, solvent, reaction time, and stirring speed, to evaluate the effects of heterogeneity and interfacial area at the water–monomer interface. The results clearly indicate that the “on-water” interface plays a significant role in increasing the polymer molecular weight. Specifically, the polymerizations of 2-methoxyethyl and 2-ethoxyethyl methacrylates in water/methanol mixtures at specific solvent volume ratios resulted in a bimodal molecular weight distribution, in which the higher-molecular-weight fraction increased as the polymerization proceeded. This polymerization behavior is attributed to a transition of the reaction medium from a homogeneous to a heterogeneous phase during polymerization, demonstrating the “on-water” interfacial effects.

## Graphical abstract

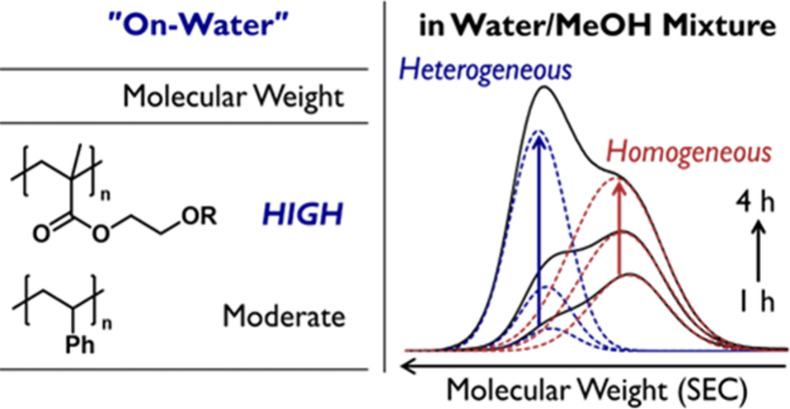

## Introduction

The use of water as a solvent offers advantages in terms of availability, low cost, environmental friendliness, and safety. Recently, particular attention has been paid to the water–organic interface, known as “on-water”, because it can significantly accelerate reaction rates.^1–9^ Sharpless et al. first introduced the concept of “on-water” and reported that significant rate enhancement was observed in a heterogeneous water–MeOH system compared to a homogeneous water/MeOH mixture.^10^ Since then, extensive mechanistic studies have been conducted, leading to various explanations, including transition-state stabilization via hydrogen bonding with dangling O–H group,^11^ enhanced reactivity due to the high acidity of interfacial water,^12^ and partial polarization at the interface arising from the orientation of water molecules.^7^ Additionally, the effects of the interfacial area by varying the stirring rate,^13^ hydrodynamic effects using microfluid devices,^14,15^ and hydrogen-bonding analyzed via ^13^C nuclear magnetic resonance (NMR) spectroscopy^16^ have been investigated.

Various polymerization methods, such as interfacial polycondensation and emulsion, suspension, and dispersion radical polymerizations, involve interfaces between aqueous and organic phases;^4^ however, polymerization studies focusing on the above-mentioned “on-water” concept have remained largely unexplored. Notably, surface-initiated Cu(0)-mediated controlled radical polymerization was remarkably accelerated under “on-water” conditions.^17^ Cu(0)-mediated polymerizations of methacrylates in water–acetonitrile or water–alcohol mixtures were also accelerated with increasing stirring speed^18^ and water content.^19^ Previously, we reported that Lewis pair radical polymerization of methacrylates and other polar vinyl monomers proceeded most efficiently under “on-water” conditions.^20,21^ In this system, the polymerization was initiated through the combination of PPh_3_ in the monomer phase and Cu­(OTf)_2_ or Fe­(OTf)_3_ in the aqueous phase. Additionally, anionic polymerization of epoxides was also accelerated at the aqueous–toluene interface.^22,23^

These recent reports demonstrate that polymerization can be accelerated under “on-water” conditions in several specific cases; however, it remains unclear whether this effect is general for conventional free radical polymerization of polar vinyl monomers. Since the molecular weight of polymers obtained via free radical polymerizations depends on various factors, including the rate coefficients of initiation, propagation, termination, and chain transfer, as well as the concentrations of monomer and initiator, and the viscosity of the polymerization mixture.^24,25^ It is well-known that a highly viscous reaction medium reduces the bimolecular termination rate, resulting in high molecular weights (Trommsdorff effect).^26–30^ We hypothesized that the “on-water” interface, in addition to these factors, influences free radical polymerization. In this study, we experimentally investigated this hypothesis using methacrylates and styrene as monomers. By varying the solvent types, employing both heterogeneous and homogeneous systems, and controlling the interfacial area via stirring speed, we demonstrate that higher-molecular-weight polymers are formed at the “on-water” interface.

## Results and Discussion

To examine the effects of “on-water” conditions on free radical polymerization, 2-methoxyethyl methacrylate (MEMA) and styrene were selected as model monomers. Styrene was used for comparison because it does not undergo hydrogen-bonding activation. The polymerization solutions were prepared via three pump-thaw cycles, and the polymerizations were conducted under high-vacuum conditions. Polymerization at elevated temperatures (e.g., 60 °C) led to boiling of the monomer and/or solvents under high-vacuum conditions; therefore, the polymerizations were carried out at 30 or 40 °C using 2,2’-azobis­(4-methoxy-2,4-dimethylvaleronitrile) (V-70) as the initiator. Table 1 summarizes the results of the radical polymerizations of MEMA (2.0 mmol, 0.29 mL) and styrene (2.0 mmol, 0.23 mL) conducted in various solvents (0.1 mL), including MeOH, *N*,*N*-dimethylformamide (DMF), 1,4-dioxane, and toluene (SEC chromatograms for Figure S1A,B). Systems showing phase separation after stirring at 30 °C and standing without agitation were defined as heterogeneous in this study. When water was employed as the solvent (entries 1 and 6), both MEMA and styrene polymerizations proceeded under heterogeneous conditions, whereas polymerization in organic solvents remained homogeneous throughout the reaction (entries 2–5 and 7–10). Therefore, the polymerizations using water as a solvent were regarded as an “on-water” system. Comparing the MEMA polymerization results in various solvents (entries 1–5), the number-average molecular weight (*M*_n_) and the molecular weight distribution (*Đ*) of poly­(MEMA) obtained “on-water” were distinctively higher than those obtained in the organic solvents, while showing comparable conversions. The monomer concentrations in the organic phase where polymerization occurred were not strictly identical between “on-water” and organic solvent systems; however, the influence of these concentration differences was limited because the polymerizations were conducted under a sufficiently high monomer volume fraction. In contrast, styrene polymerization showed no significant differences in *M*_n_ and *Đ* values of the resulting polystyrene between organic solvent systems and the “on-water” system (entries 6–10). When the polymerizations of MEMA and styrene were similarly conducted using 0.5 mL of solvents, the MEMA polymerization “on-water” afforded distinctively high-molecular-weight polymers, while no significant differences were observed in the other systems (Table S1, SEC chromatograms for Figure S1C,D).

**Table 1:** Solvent Effects on the Free Radical Polymerization of MEMA and Styrene^a^

				conv.^c^	*M*_n_ ^d^	
	monomer	solvent^b^	polymerization solution	%	kg/mol	*Đ* ^d^
1	MEMA	water	heterogeneous	47	200	2.70
2	MEMA	MeOH	homogeneous	41	110	2.27
3	MEMA	DMF	homogeneous	42	97	2.21
4	MEMA	1,4-dioxane	homogeneous	39	110	2.14
5	MEMA	toluene	homogeneous	42	110	2.09
6	styrene	water	heterogeneous	26	6.8	1.68
7	styrene	MeOH	homogeneous	34	7.7	1.61
8	styrene	DMF	homogeneous	20	5.2	1.50
9	styrene	1,4-dioxane	homogeneous	25	5.8	1.60
10	styrene	toluene	homogeneous	32	6.0	1.57

a Conditions of entries 1–5: MEMA (2.0 mmol, 0.29 mL), V-70 (1.0 mol %), solvent (0.10 mL), 30 °C, 2 h, under vacuum. Conditions of entries 6–10: St (2.0 mmol, 0.23 mL), V-70 (2.0 mol %), solvent (0.10 mL), 40 °C, 4 h, under vacuum.

b DMF: *N*,*N*-dimethylformamide.

c ^1^H NMR.

d SEC (entries 1–5: DMF eluent using poly­(methyl methacrylate) standards, entries 6–10: CHCl_3_ eluent using polystyrene standards).

We then examined the hydrogen-bonding capability of MeOH with the carbonyl group of methacrylate monomers. The ^13^C NMR spectrum of a mixture of MEMA and MeOH in CDCl_3_ exhibited apparent downfield shifts of both the β-carbon and the carbonyl carbon in MEMA, whereas the α-carbon showed an upfield shift (Figure S2A). These results indicate that hydrogen bonding between the carbonyl group of MEMA and MeOH decreased the electron density at the β-carbon and carbonyl carbon. Although direct analysis of the “on-water” interface is difficult, a CDCl_3_ solution of MEMA saturated with water was analyzed by ^13^C NMR (Figure S2B). Slight but discernible chemical shift changes, similar to those observed in the MeOH solution, were detected, indicating that water dissolved in the homogeneous phase is also capable of hydrogen bonding. Given that both water and MeOH are capable of hydrogen bonding with MEMA in the homogeneous phase, the significant differences in polymer molecular weights between “on-water” and organic solvent conditions (Table 1) cannot be explained by monomer activation via hydrogen bonding in the homogeneous phase.

To investigate the effects of the water–monomer interface on the polymerization in greater detail, MEMA polymerizations were carried out in water/MeOH mixtures with systematically varied mixed ratios in small increments. The SEC chromatograms, as well as *M*_n_ and *Đ* values of the obtained poly­(MEMA), are shown in Figure 1A and Tables S2 and S3, respectively. Polymers obtained at higher water volume ratios (water/MeOH > 54/46, v/v; blue SEC curves in Figure 1A) exhibited higher molecular weights than those obtained at lower water ratios (water/MeOH < 44/56, v/v; black SEC curves in Figure 1A). Notably, bimodal SEC chromatograms were observed at the water/MeOH volume ratios ranging from 46/54 to 52/48 (Figure 1A; see also Gaussian deconvolution in Figure S3), suggesting a correlation with the onset of phase heterogeneity. Under these conditions, the mixtures transitioned from homogeneous to heterogeneous with increasing water content. Polymerization solutions with water/MeOH ratios of 54/46 or higher were heterogeneous before the reaction, whereas those with ratios of 52/48 or lower were homogeneous. The intermediary states between the homogeneous and heterogeneous phases were confirmed by microscopic analysis of solutions containing uranine, a fluorescent dye, in the absence of agitation (Figure S4), where observed water droplets were significantly larger than those present under stirring conditions.

**Figure 1:**
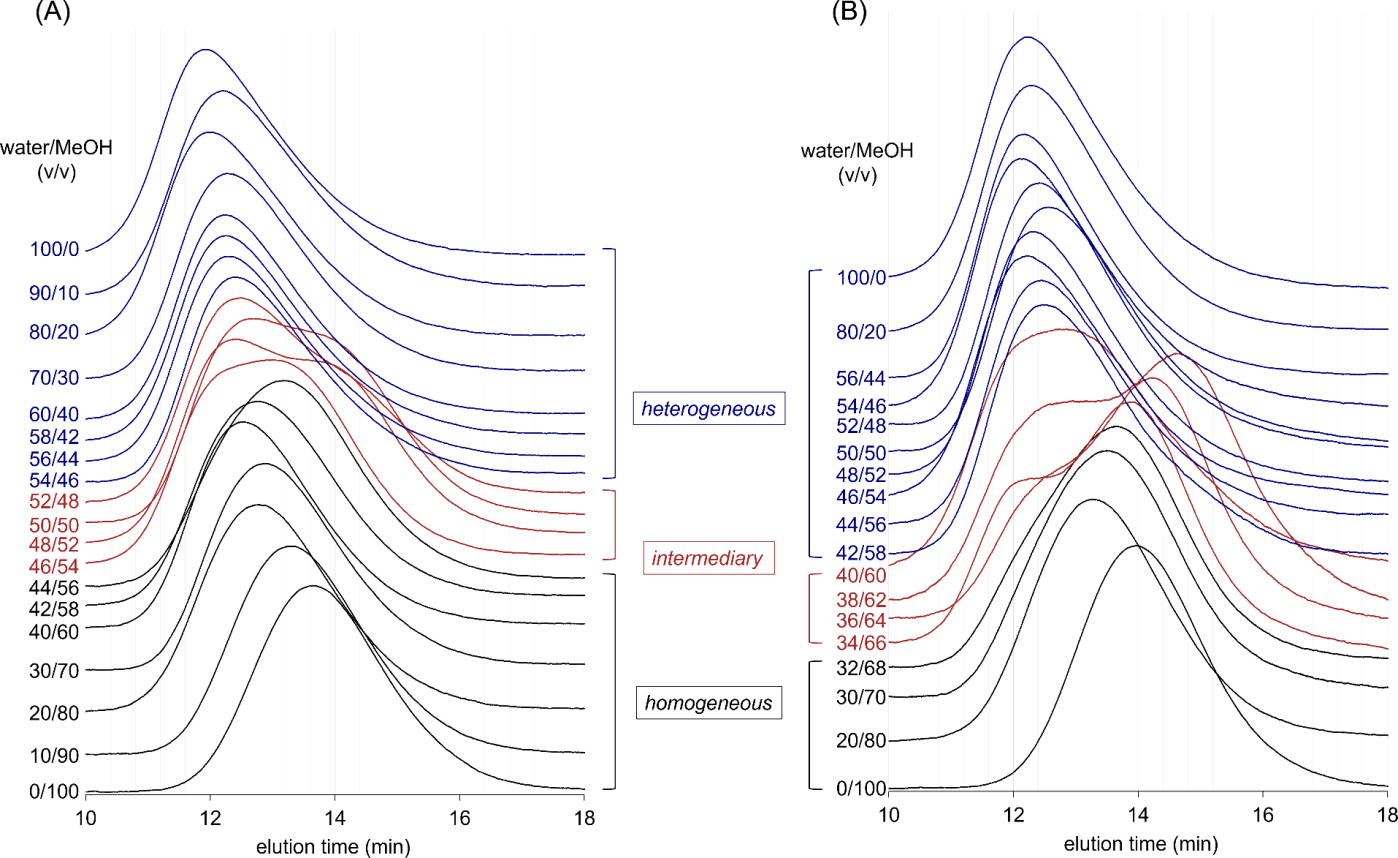
SEC chromatograms of (A) poly­(MEMA) and (B) poly­(EEMA) obtained in water/MeOH mixtures with various volume ratios. The polymerizations were carried out under conditions similar to entries 1–5 in Table S1.

To examine the effects of the monomer hydrophilicity, similar experiments were conducted using 2-ethoxyethyl methacrylate (EEMA, water solubility of 17 g/L at 20 °C), which is slightly less hydrophilic than MEMA (water solubility of 31 g/L at 20 °C). As expected, systems with water/MeOH ratios of 42/58 or higher were heterogeneous, resulting in higher molecular weights, whereas those of 32/68 or lower were homogeneous, yielding lower molecular weights. Bimodal SEC chromatograms were observed at ratios ranging from 34/66 to 40/60 (Figure 1B; see also Gaussian deconvolution in Figure S5, and microscopic analysis in Figure S6). The water fractions at these transition points were lower than those for MEMA, reflecting the lower hydrophilicity of EEMA. These results indicate that the bimodal SEC distributions arise under initially homogeneous conditions close to the phase boundary with the heterogeneous state.

To investigate the origin of the bimodal SEC chromatograms, MEMA polymerizations were carried out at a water/MeOH ratio of 50/50 for 0.5, 1, 2, and 4 h, and SEC chromatograms were obtained at different MEMA conversions (Figure 2A; see also Gaussian deconvolution in Figure S7). At 10% conversion, a low-molecular-weight polymer with a small high-molecular-weight fraction was observed. As the conversion increased to 25% and 54%, the high-molecular-weight fraction increased markedly. The low-molecular-weight polymer is initially formed because the polymerization system is homogeneous at the early stage of the polymerization. As the polymerization proceeds, increasing the fraction of the polymer, which is more hydrophobic than the monomer, induces a transition from homogeneous to heterogeneous. This phase separation generates interfaces between the organic phase (monomer and polymer) and water, thereby promoting the formation of high-molecular-weight polymer. Similar polymerization behavior was observed for EEMA in a water/MeOH ratio of 38/62 (Figure 2(B); see also Gaussian deconvolution in Figure S8).

**Figure 2:**
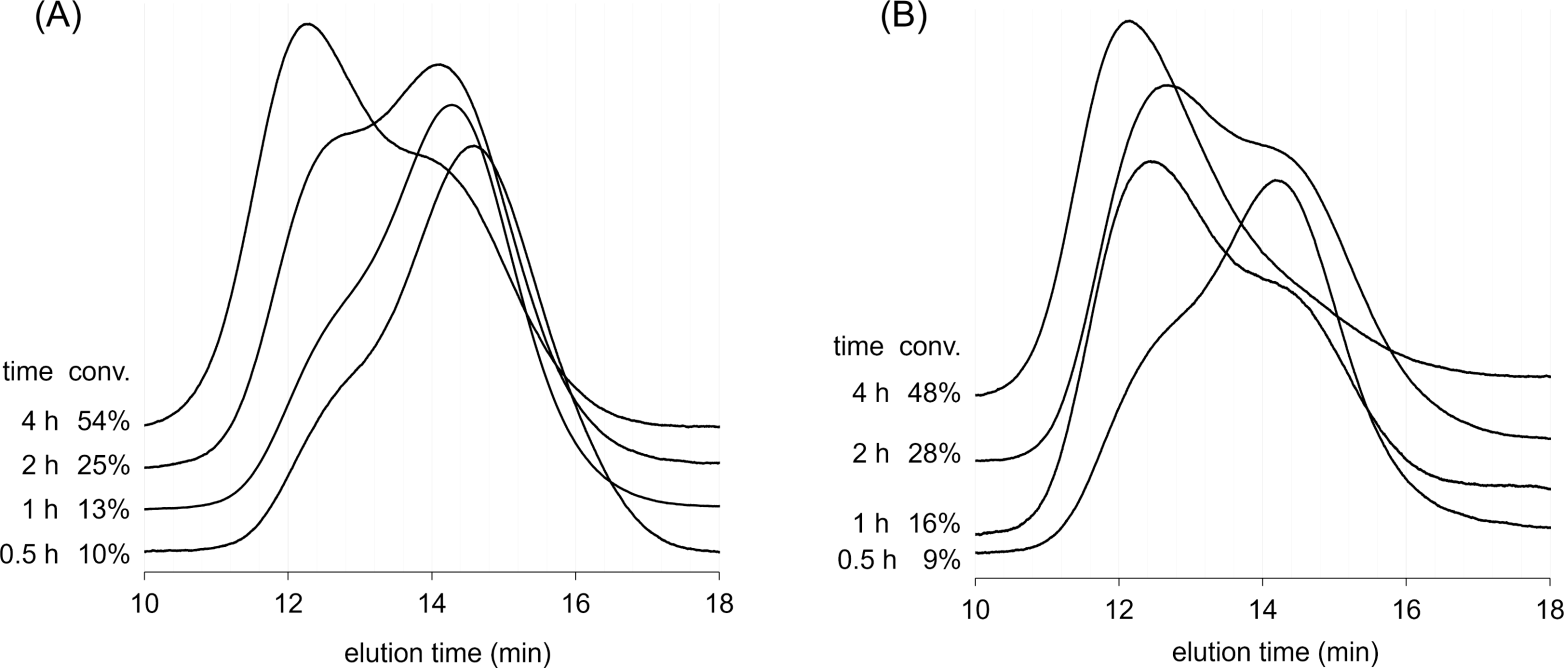
SEC chromatograms of (A) poly­(MEMA) obtained in a water/MeOH mixture (50/50, v/v) and (B) poly­(EEMA) obtained in a water/MeOH mixture of (38/62, v/v) at different monomer conversions. The polymerizations were carried out under conditions similar to entries 1–5 in Table S1.

The above results indicate that phase separation leads to the formation of high-molecular-weight polymers. However, the “on-water” system can essentially be regarded as a bulk system containing added water. The polymerizations in bulk (Figure 3A) and “on-water” (Figure 3B) were then compared at various stirring speeds (100–1500 rpm). In bulk polymerizations, higher-molecular-weight polymers were obtained at lower stirring speeds (100–500 rpm), whereas the molecular weights apparently decreased at higher stirring speeds (1000 and 1500 rpm) (Figure 3A). Although local viscosity could not be directly measured, slower polymer diffusion at lower stirring speeds may suppress bimolecular termination in a manner analogous to the Trommsdorff effect.^30^ In contrast, high-molecular-weight polymers were obtained in the “on-water” system even under the high-stirring-speed conditions (Figure 3B), where water droplets were highly dispersed. Therefore, this clear difference between “on-water” and bulk polymerizations supports the conclusion that the monomer–water interface, as well as the local viscosity, play critical roles in the formation of high-molecular-weight polymers.

**Figure 3:**
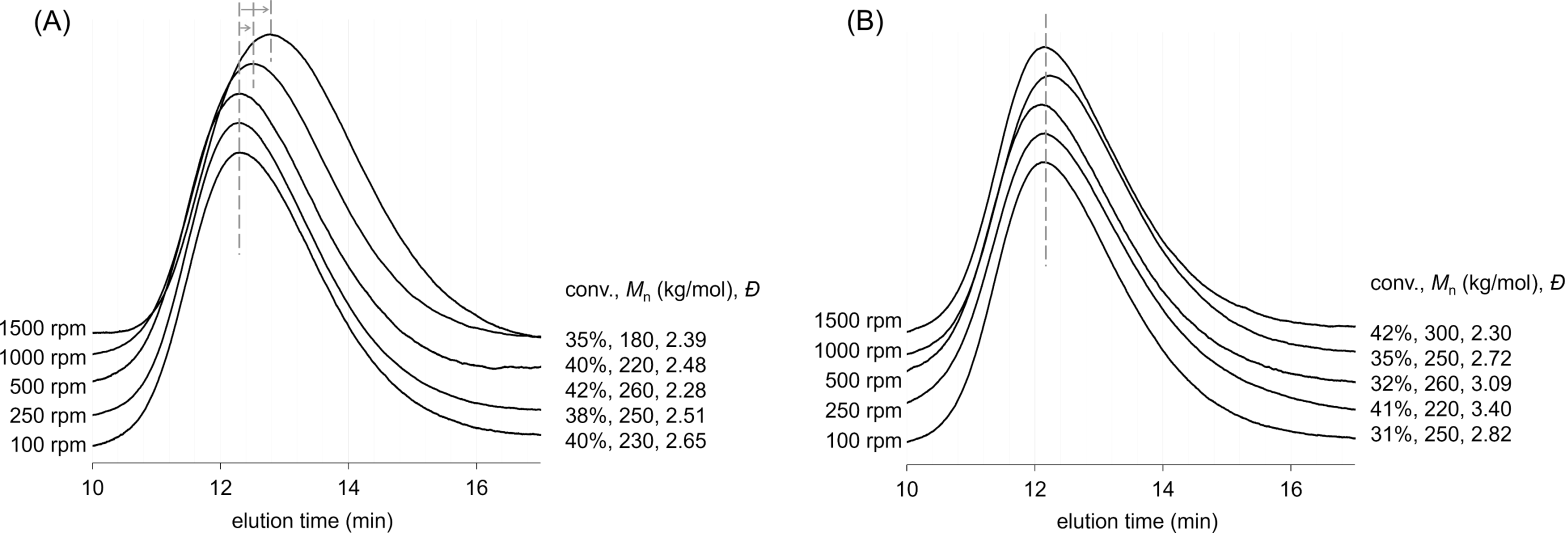
SEC chromatograms of poly­(MEMA) obtained under various stirring speeds (A) in bulk and (B) “on-water”. The polymerizations were carried out under conditions similar to entry 1 in Table S1.

## Conclusion

We investigated the effects of “on-water” conditions on free radical polymerization. Polymerization of MEMA under “on-water” conditions afforded the highest molecular weights, whereas no significant “on-water” effect was observed for styrene polymerization. When methanol was used as the solvent, higher-molecular-weight polymers were not obtained, despite the apparent hydrogen bonding between MEMA and methanol. Polymerizations were further examined in water/methanol mixtures with systematically varied volume ratios, where higher molecular weights were obtained under heterogeneous conditions. Notably, bimodal molecular weight distributions were observed under intermediary states between homogeneous and heterogeneous phases. The molecular weight distributions as a function of conversion indicated that these bimodal distributions are attributable to the onset of phase separation during the polymerization. Comparison of bulk and “on-water” polymerizations at varying stirring speeds revealed that the “on-water” effect becomes more pronounced at larger interfacial areas, indicating that it cannot be explained solely by the Trommsdorff effect and chain transfer events. Therefore, it is reasonable to conclude that activation of the monomer carbonyl group through hydrogen bonding with dangling O–H groups and/or partial polarization at the interface may increase the propagation rate, thereby leading to higher molecular weights. Free radical polymerization in/on aqueous media is widely used on an industrial scale, and reproducible control of molecular weight and its distribution is of great importance. This study indicates that the heterogeneity of the reaction system strongly influences molecular weight and its distribution. Since conventional suspension polymerization systems also involve monomer–water interfaces, the “on-water” effects identified here may likewise influence industrial processes.

## Supplementary Material



## Notes

Experimental section, SEC chromatograms, supplementary polymerization Table, ^13^C NMR spectra, and images of the polymerization solutions (PDF)
