# Supplementary material for: Effects of “On-Water” on the Free Radical Polymerization of Methacrylates
Source: Langmuir. 2026 Jun 9;42(24):17615–9. doi: 10.1021/acs.langmuir.6c01890 (PMC13296471; doi:10.1021/acs.langmuir.6c01890)
Supplement: Supplementary file 1 [file la6c01890_si_001.pdf]

*Supporting information for*

# Effects of "On-Water" on Free Radical Polymerization of Methacrylates

Yusaku Kambe and Shin-ichi Matsuoka*

Division of Soft Materials, Department of Engineering, Graduate School of Engineering,

Nagoya Institute of Technology, Gokiso-cho, Showa-ku, Nagoya, Aichi 466-8555, Japan

Corresponding Author: Shin-ichi Matsuoka

http://orcid.org/0000-0001-7488-9971

E-mail: matsuoka.shinichi@nitech.ac.jp

Tel: +81-52-735-7254

# CONTENTS

EXPERIMENTAL SECTION........................................................................................3
Instrumentation ........................................................................................................3
Materials..................................................................................................................3
Polymerization procedure ..........................................................................................3
NMR experiments (Figure S2)......................................................................................4
Figure S1. SEC chromatograms of polymers obtained in various solvents (0.1 mL or 0.5 mL). (A) poly(MEMA) (0.1 mL), (B) polystyrene (0.1 mL), (C) poly(MEMA) (0.5 mL), and (D) polystyrene (0.5 mL), ........................................................................................................5
Table S1. Solvent effects on free radical polymerization of MEMA and styrene (solvent: 0.5 mL)[a] .6
Figure S2. $^{13}C$ NMR chemical shift changes upon addition of MeOH (A) and water (B). Carbons shifted to downfield are highlighted in red. ................................................................7
Table S2. Polymerization of MEMA in a mixture of water and MeOH with various volume ratios (Figure 1(A)). ..............................................................................................................8
Table S3. Polymerization of EEMA in a mixture of water and MeOH with various volume ratios (Figure 1(B)). ..............................................................................................................9
Figure S3. Gaussian deconvolution of the bimodal SEC chromatograms of poly(MEMA) shown in Figure 1(A)...............................................................................................................10
Figure S4. Images of the MEMA polymerization solutions (Figure 1(A)) in various water/MeOH ratios at 30 °C without agitation......................................................................................11
Figure S5. Gaussian deconvolution of the bimodal SEC chromatograms of poly(EEMA) shown in Figure 1(B). ..............................................................................................................12
Figure S6. Images of the EEMA polymerization solutions (Figure 1(B)) in various water/MeOH ratios at 30 °C without agitation .....................................................................................13
Figure S7. Gaussian deconvolution of the bimodal SEC chromatograms poly(MEMA) shown in Figure 2(A)...............................................................................................................14
Figure S8. Gaussian deconvolution of the bimodal SEC chromatograms poly(EEMA) shown in Figure 2(B). ..............................................................................................................15

EXPERIMENTAL SECTION

Instrumentation

$^1H$ NMR spectra were obtained using a Bruker Avance III HD spectrometer operating at 400 MHz for $^1H$ and 100 MHz for $^{13}C$ at 25 °C in $CDCl_3$. The molecular weights and molecular weight distributions of poly(MEMA) and poly(EEMA) were estimated by size-exclusion chromatography (SEC) equipped with a RI-4035 (JASCO) detector and two tandem LF-404 columns (Shodex) calibrated against poly(methyl methacrylate) standards using DMF/LiBr (containing 5.75 mmol/L LiBr, at 40 °C with a flow rate of 0.3 mL $min^{-1}$) as the eluent. The molecular weights and molecular weight distributions of polystyrene were estimated by SEC equipped with an RI-4035 (JASCO) detector and two tandem HK-404L columns (Shodex) calibrated using monodisperse polystyrene standards using $CHCl_3$ (at 40 °C with a flow rate of 0.3 mL $min^{-1}$) as the eluent. The solution images were taken using an Ivesta 3 microscope (Leica).

Materials

MEMA (TCI, 98%) and EEMA (TCI, >98%) were distilled from $CaH_2$ under reduced pressure and stored over molecular sieve 3Å. Styrene (TCI, >99%) was washed with 2M aqueous NaOH and brine, dried over anhydrous MgSO4, and subsequently distilled from $CaH_2$ under reduced pressure. V-70 (Wako, >95%) was recrystallized from MeOH before use. MeOH (Wako, Super Dehydrated, >99.8%) used in the polymerization solvent was stored over molecular sieve 3Å. Distilled water (Nacalai Tesque, HPLC grade) was used as received. Toluene (Kanto, >99%) and 1,4-dioxane (Kishida, >99.5%) were distilled from $CaH_2$ under reduced pressure and stored over molecular sieve 3Å. DMF (Kanto, >99%) was distilled under reduced pressure and stored over molecular sieve 3Å.

Polymerization procedure

All polymerizations were performed in a reaction tube (φ = 20 mm) equipped with a two-way stopcock under reduced pressure.

As a typical procedure for the MEMA polymerization (entry 1, Table 1), a mixture of MEMA (288 mg, 2.00 mmol, 0.29 mL), V-70 (6.2 mg, 0.020 mmol), and water (0.1 mL) in a reaction tube was degassed by three freeze–pump–thaw cycles. Polymerization was initiated by stirring the mixture at 500 rpm (unless otherwise noted) at 30±1 °C in a water bath. After 2 h, the two-way stopcock was opened to air, and 1 mL of THF containing a small amount of phenothiazine as a polymerization inhibitor was added to homogenize the mixture. An aliquot was sampled and analyzed by $^1H$ NMR. The monomer conversion was estimated from the integral ratio of vinyl protons and methylene (-$OCH_2$-) protons. The polymerization mixture was reprecipitated into $Et_2O$ to afford poly(MEMA) (120 mg) in 42% yield.

The polymerization of EEMA was performed similarly to the MEMA polymerization

described above. Poly(EEMA) was obtained by reprecipitation into a mixture of $Et_2O$/hexane (1:2, v/v).

The polymerization of styrene was performed similarly to the MEMA polymerization described above, except that the polymerization time and temperature were 4 h and 40 °C, respectively. The monomer conversion was estimated from the integral ratio of the vinyl protons to the phenyl protons. The polystyrene was obtained by reprecipitation into MeOH.

NMR experiments (Figure S2)

A mixture of MEMA (0.10 mmol, 14 mg), $CDCl_3$ (0.50 mL), and water (0.10 mL) was stirred overnight at room temperature. The solution was then allowed to stand, and the $CDCl_3$ layer was analyzed by $^1H$ and $^{13}C$ NMR spectroscopy.

MeOH (30 μL), MEMA (0.10 mmol, 14 mg), and $CDCl_3$ (0.50 mL) were mixed and subjected to $^{13}C$ NMR spectroscopy.

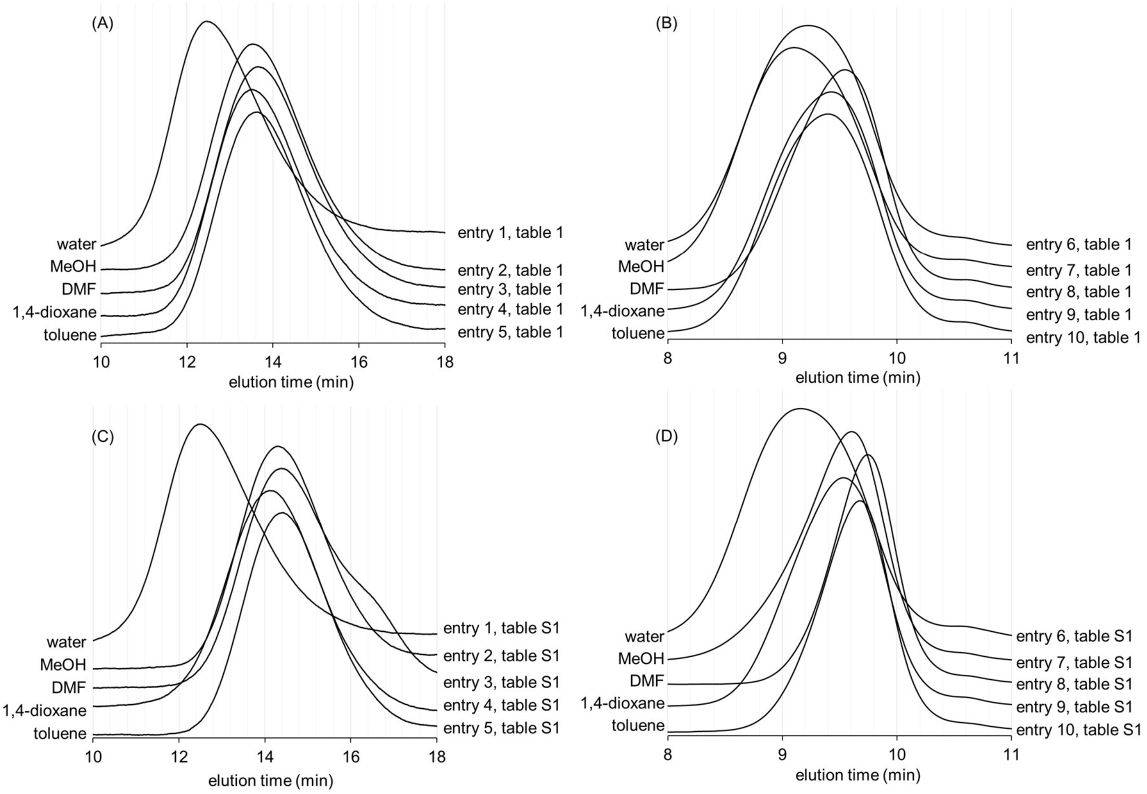


Figure S1. SEC chromatograms of polymers obtained in various solvents (0.1 mL or 0.5 mL). (A) poly(MEMA) (0.1 mL), (B) polystyrene (0.1 mL), (C) poly(MEMA) (0.5 mL), and (D) polystyrene (0.5 mL),

Table S1. Solvent effects on free radical polymerization of MEMA and styrene (solvent: 0.5 mL)[a]

| entry | monomer | solvent[b] | polymerization solution | conv.[c] (%) % | $M_n$[d] kg/mol | $Đ$[d] |
|---|---|---|---|---|---|---|
| 1 | MEMA | water | heterogeneous | 49 | 210 | 2.76 |
| 2 | MEMA | MeOH | homogeneous | 31 | 68 | 1.86 |
| 3 | MEMA | DMF | homogeneous | 36 | 43 | 2.53 |
| 4 | MEMA | 1,4-dioxane | homogeneous | 12 | 78 | 2.25 |
| 5 | MEMA | toluene | homogeneous | 24 | 61 | 1.92 |
| 6 | St | water | heterogeneous | 29 | 6.5 | 1.82 |
| 7 | St | $CH_3OH$ | homogeneous | 15 | 5.1 | 1.56 |
| 8 | St | DMF | homogeneous | 8 | 3.8 | 1.33 |
| 9 | St | 1,4-dioxane | homogeneous | 22 | 5.1 | 1.50 |
| 10 | St | toluene | homogeneous | 13 | 4.3 | 1.36 |

[a]conditions of entries 1–5: MEMA (2.0 mmol, 0.29 mL), V-70 (1.0 mol%), solvent (0.50 mL), 30 °C, 2 h, under vacuum. Conditions of entries 6–10: St (2.0 mmol, 0.23 mL), V-70 (2.0 mol%), solvent (0.50 mL), 40 °C, 4 h, under vacuum. [b]DMF: *N*,*N*-dimethylformamide. [c]$^1$H NMR. [d]SEC (entries 1–5: DMF/LiBr eluent using poly(methyl methacrylate) standards, entries 6–10: $CHCl_3$ eluent using polystyrene standards).

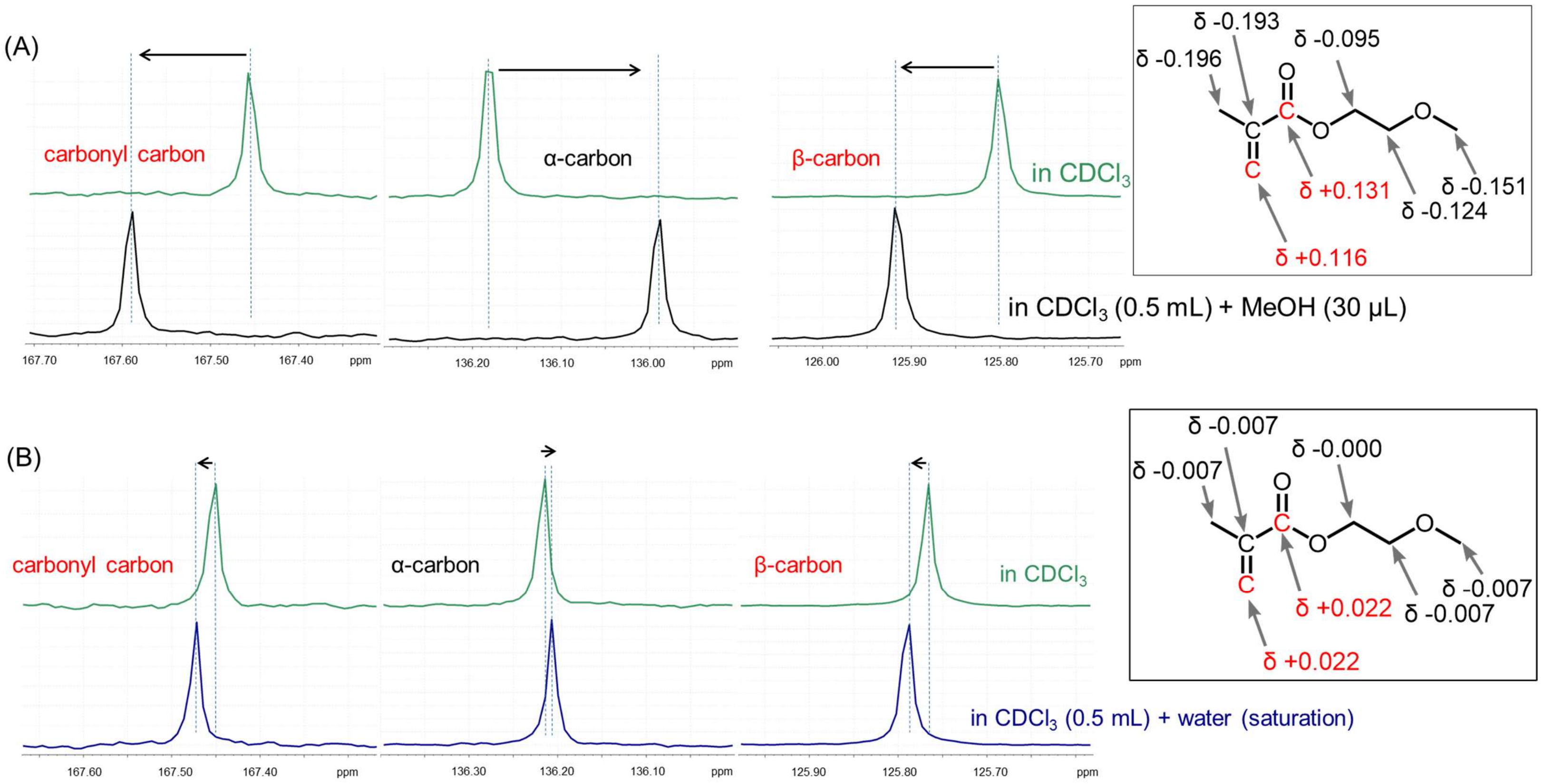


Figure S2. $^{13}$C NMR chemical shift changes upon addition of MeOH (A) and water (B). Carbons shifted to downfield are highlighted in red.

Table S2. Polymerization of MEMA in a mixture of water and MeOH with various volume ratios (Figure 1(A)).

| entry | water/MeOH | conv. [b] | $M_n$ [c] | $Đ$ [c] |
|---|---|---|---|---|
| | v/v | % | kg/mol | |
| 1 | 0/100 | 28 | 110 | 2.17 |
| 2 | 10/90 | 38 | 140 | 2.18 |
| 3 | 20/80 | 41 | 210 | 2.31 |
| 4 | 30/70 | 42 | 180 | 2.25 |
| 5 | 40/60 | 43 | 240 | 2.17 |
| 6 | 42/58 | 43 | 200 | 2.40 |
| 7 | 44/56 | 42 | 170 | 2.44 |
| 8 | 46/54 | 34 | 200 | 2.61 |
| 9 | 48/52 | 38 | 150 | 3.10 |
| 10 | 50/50 | 33 | 140 | 2.89 |
| 11 | 52/48 | 35 | 160 | 3.03 |
| 12 | 54/46 | 35 | 210 | 2.52 |
| 13 | 56/44 | 31 | 240 | 2.54 |
| 14 | 58/42 | 34 | 250 | 2.59 |
| 15 | 60/40 | 32 | 260 | 2.50 |
| 16 | 70/30 | 45 | 260 | 2.45 |
| 17 | 80/20 | 55 | 310 | 2.56 |
| 18 | 90/10 | 47 | 260 | 2.62 |
| 19 | 100/0 | 46 | 340 | 2.54 |

[a] MEMA (2.0 mmol, 0.29 mL), V-70 (1.0 mol%), total solvent volume (0.50 mL), 30 °C, 2 h, under vacuum.
[b] calculated by $^1$H NMR.
[c] SEC (DMF eluent using poly(methyl methacrylate) stanards).

Table S3. Polymerization of EEMA in a mixture of water and MeOH with various volume ratios (Figure 1(B)).

| entry | water/MeOH | conv. [b] | $M_n$ [c] | $Đ$ [c] |
|---|---|---|---|---|
| | v/v | % | kg/mol | |
| 1 | 0/100 | 30 | 83 | 2.11 |
| 2 | 20/80 | 47 | 130 | 2.44 |
| 3 | 30/70 | 40 | 97 | 2.71 |
| 4 | 32/68 | 29 | 98 | 2.85 |
| 5 | 34/66 | 24 | 93 | 3.95 |
| 6 | 36/64 | 26 | 76 | 3.40 |
| 7 | 38/62 | 21 | 61 | 4.79 |
| 8 | 40/60 | 15 | 110 | 4.63 |
| 9 | 42/58 | 34 | 150 | 2.98 |
| 10 | 44/56 | 34 | 160 | 2.94 |
| 11 | 46/54 | 23 | 150 | 3.82 |
| 12 | 48/52 | 38 | 170 | 3.00 |
| 13 | 50/50 | 40 | 120 | 3.44 |
| 14 | 52/48 | 39 | 170 | 2.75 |
| 15 | 54/46 | 18 | 170 | 3.31 |
| 16 | 56/44 | 34 | 220 | 2.55 |
| 17 | 80/20 | 52 | 250 | 2.44 |
| 18 | 100/0 | 40 | 230 | 2.86 |

[a] EEMA (2.0 mmol, 0.33 mL), V-70 (1.0 mol%), total solvent volume (0.50 mL), 30 °C, 2 h, under vacuum.
[b] calculated by $^1H$ NMR.
[c] SEC (DMF eluent using poly(methyl methacrylate) stanards).

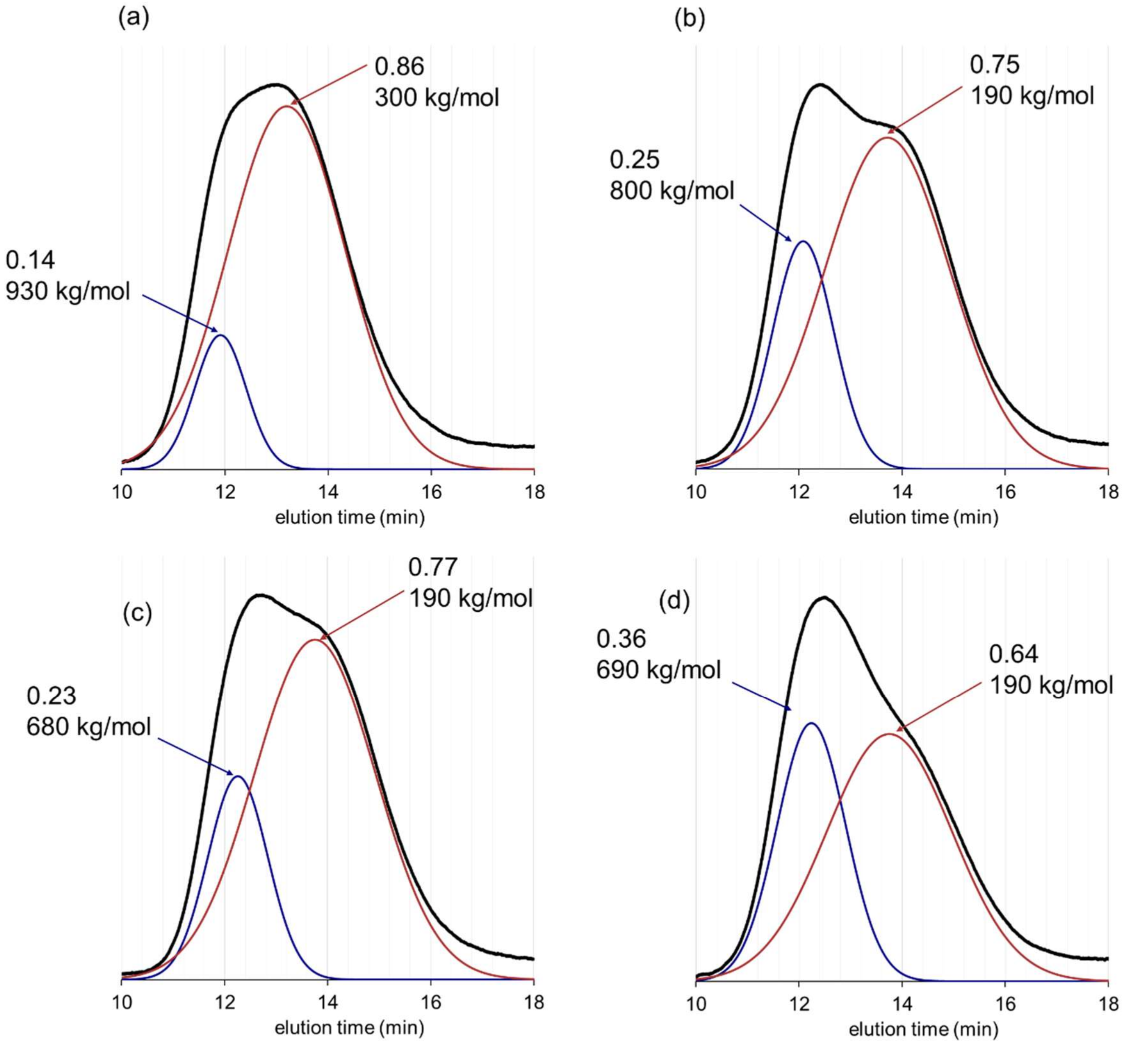


Figure S3. Gaussian deconvolution of the bimodal SEC chromatograms of poly(MEMA) shown in Figure 1(A).

The peak area ratio and molecular weight at the peak top ($M_p$) are indicated. The solvent volume ratios of water/MeOH were as follows: (a) 46/54, (b) 48/52, (c) 50/50, and (d) 52/48.

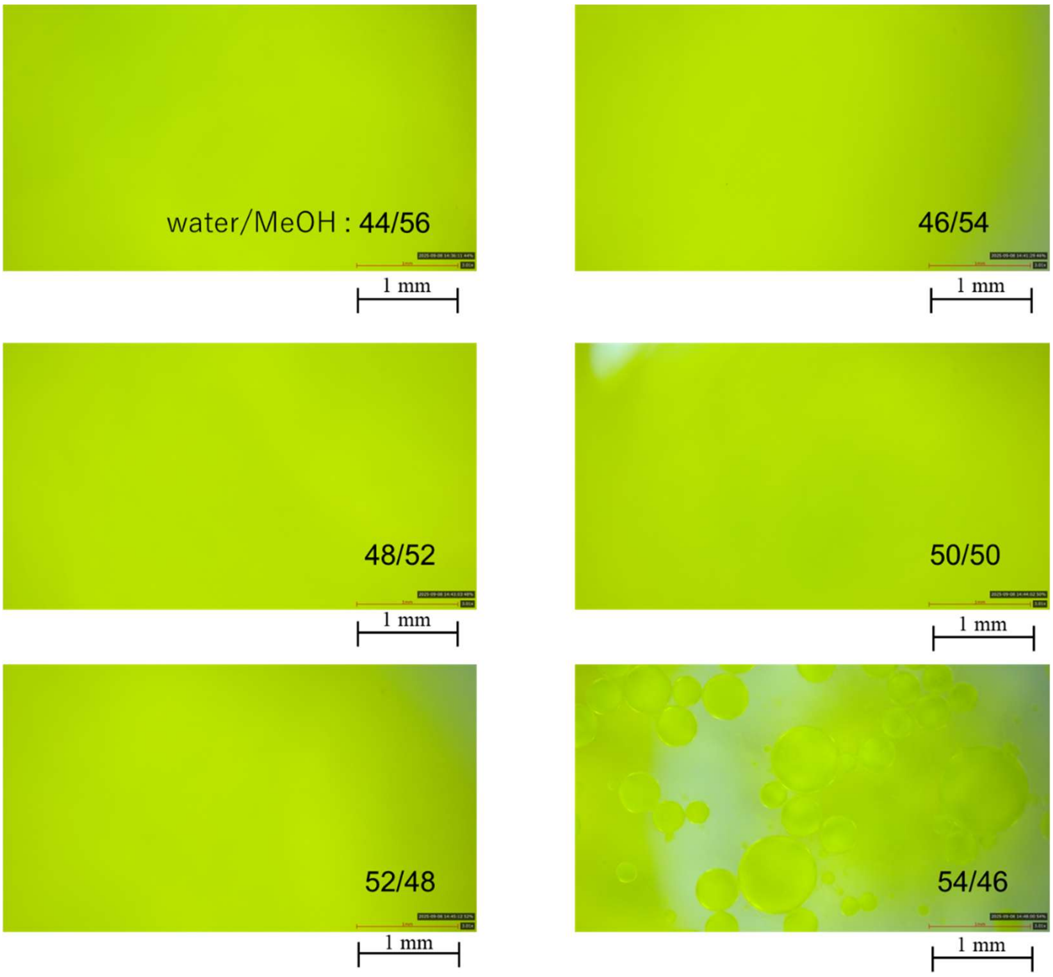


Figure S4. Images of the MEMA polymerization solutions (Figure 1(A)) in various water/MeOH ratios at 30 °C without agitation

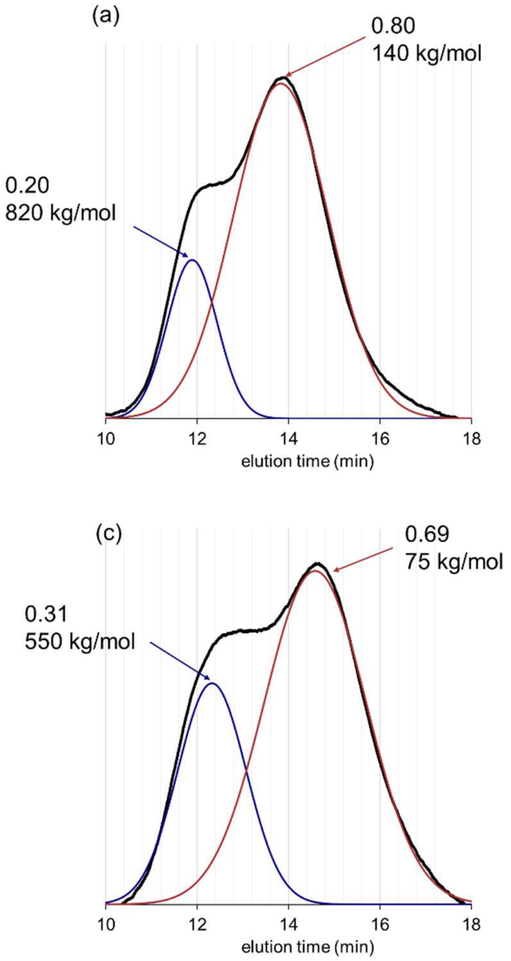


Figure S5. Gaussian deconvolution of the bimodal SEC chromatograms of poly(EEMA) shown in Figure 1(B).
The peak area ratio and molecular weight at the peak top ($M_p$) are indicated. The solvent volume ratios of water/MeOH were as follows: (a) 34/66, (b) 36/64, and (c) 38/62.

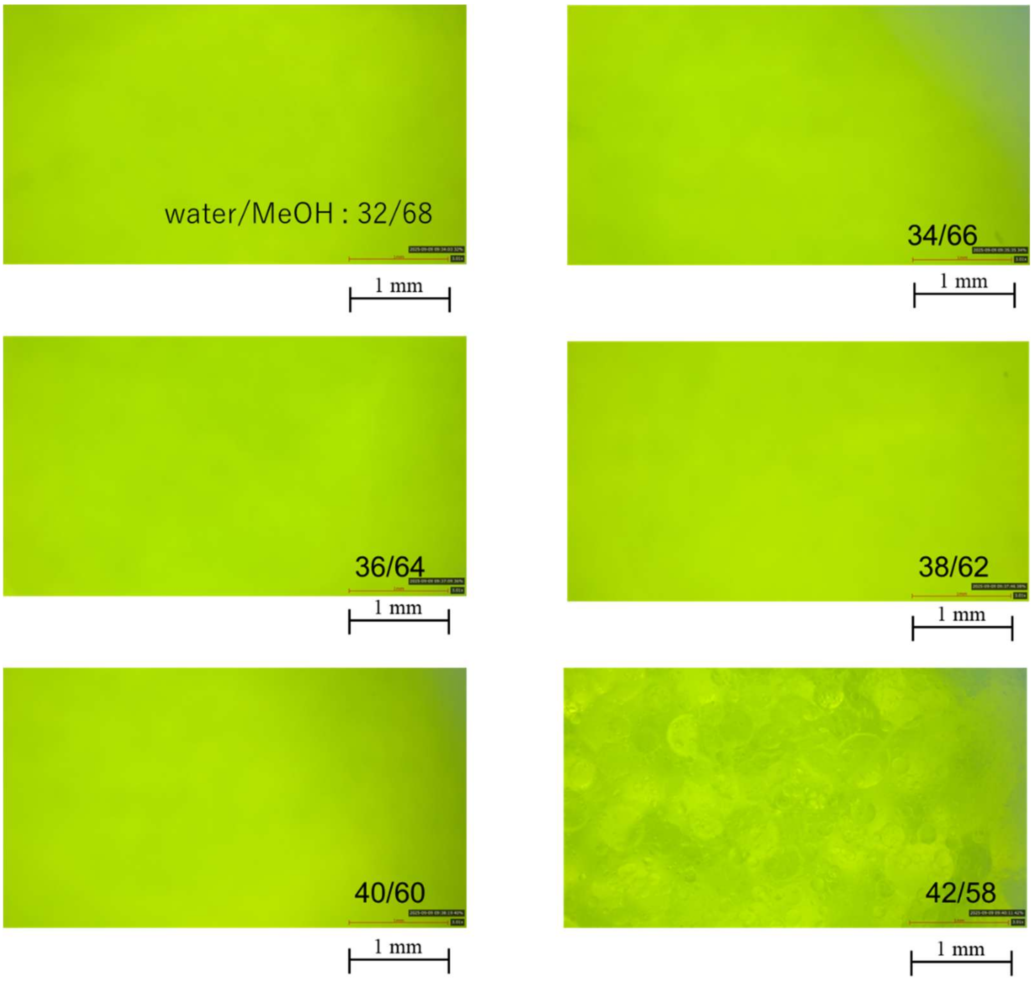


Figure S6. Images of the EEMA polymerization solutions (Figure 1(B)) in various water/MeOH ratios at 30 °C without agitation

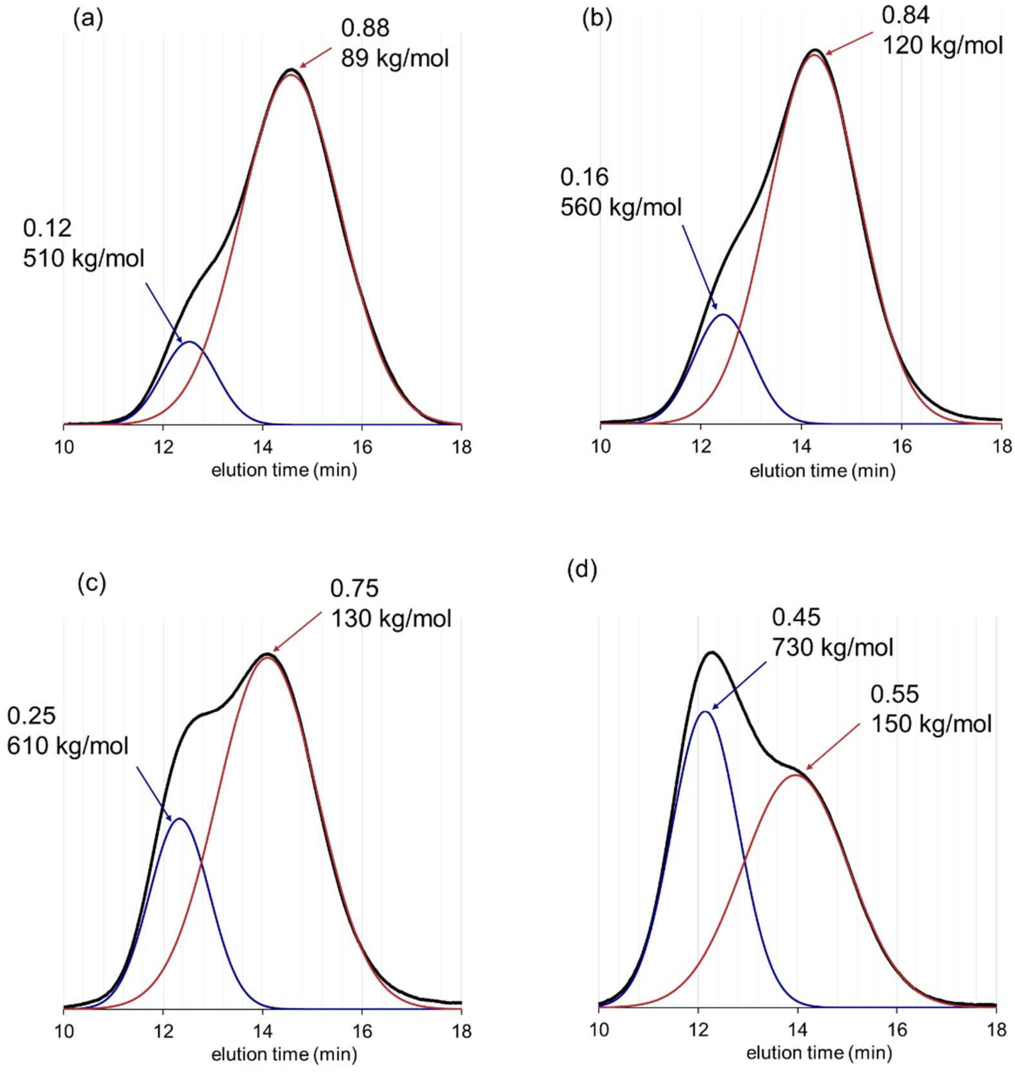


Figure S7. Gaussian deconvolution of the bimodal SEC chromatograms poly(MEMA) shown in Figure 2(A).
The peak area ratio and molecular weight at the peak top ($M_p$) are indicated. The corresponding polymerization time and conversion were as follows: (a) 0.5 h, 10%; (b) 1 h, 13%; (c) 2 h, 25%; and (d) 4 h, 54%.

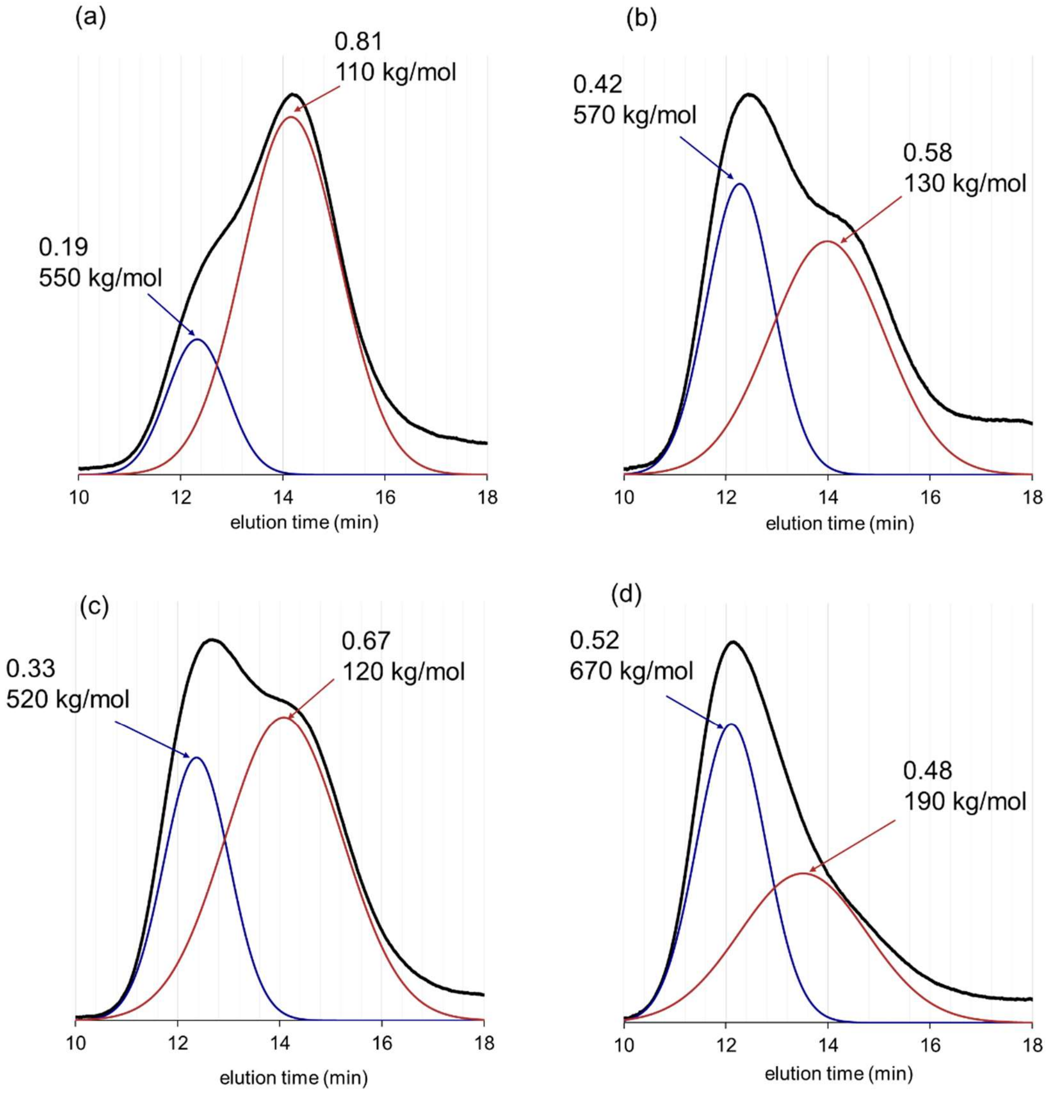


Figure S8. Gaussian deconvolution of the bimodal SEC chromatograms poly(EEMA) shown in Figure 2(B).

The peak area ratio and molecular weight at the peak top ($M_p$) are indicated. The corresponding polymerization time and conversion were as follows: (a) 0.5 h, 9%; (b) 1 h, 16%; (c) 2 h, 28%; and (d) 4 h, 48%.
